# Performance of large language models and prompt engineering strategies for data extraction in systematic reviews

**DOI:** 10.3389/fdgth.2026.1799623

**Published:** 2026-04-29

**Authors:** Takehiko Oami, Yohei Okada, Kenjiro Maeda, Taka-aki Nakada

**Affiliations:** 1 Department of Emergency and Critical Care Medicine, Chiba University Graduate School of Medicine, Chiba, Japan; 2 Department of Preventive Services, Kyoto University Graduate School of Medicine, Kyoto, Japan

**Keywords:** clinical practice guidelines, data extraction, large language model, sepsis, systematic review

## Abstract

**Background:**

Systematic reviews depend on manual data extraction and synthesis, which are time-consuming and prone to human error. Although large language models (LLMs) have the potential to automate parts of this process, their accuracy, reproducibility, and efficiency across different models and prompt strategies remain insufficiently characterized.

**Methods:**

This study evaluated the performance of three LLMs, including ChatGPT-4o, Claude 3 Sonnet, and Gemini 1.5 Pro, for data extraction from trials addressing five clinical questions (CQs) in the Japanese Clinical Practice Guidelines for the Management of Sepsis and Septic Shock 2024 (J-SSCG 2024). Using portable document format files of eligible studies, LLMs extracted predefined background characteristics and clinical outcomes. Outputs generated using an original prompt were compared with those produced using chain-of-thought and self-reflection (SR) prompt strategies. Two independent reviewers assessed accuracy against a reference standard derived from manual extraction by the guideline members. Inter-session consistency across three sessions and processing time were also evaluated.

**Results:**

For background data extraction, mean no-error proportions ranged from 81.6% (ChatGPT-4o) to 92.4% (Claude 3 Sonnet) across models. For outcome data extraction, mean no-error proportions ranged from 27.8% (Gemini 1.5 Pro) to 80.7% (Claude 3 Sonnet). Missing or incorrect values accounted for most extraction errors, whereas fabricated outputs were relatively uncommon. Prompt engineering strategies resulted in only modest changes in extraction accuracy across models. Inter-session consistency ranged from 76.3% (ChatGPT-4o) to 91.3% (Gemini 1.5 Pro) for background data extraction and from 44.8% (ChatGPT-4o) to 65.6% (Claude 3 Sonnet) for outcome data extraction. Mean processing times ranged from 29.2 to 39.7 s per article for background data extraction and from 19.3 to 46.3 s for outcome data extraction using standard prompts. When SR prompts were used, processing times increased to 59.0 to 97.7 s for background data extraction and to 52.7 to 107.1 s for outcome data extraction.

**Conclusions:**

LLMs can reliably support background data extraction in systematic reviews. However, outcome data extraction remains challenging, emphasizing the continued need for human oversight. Extraction performance varied across models and prompt engineering strategies.

**Clinical Trial Registration**: The study was registered in the University Hospital Medical Information Network (UMIN) clinical trials registry, identifier (UMIN000054461).

## Background

Systematic reviews are fundamental to synthesizing existing evidence and informing decision-making in healthcare (1, 2). However, conventional systematic review workflows rely heavily on manual screening and data extraction, rendering them labor-intensive, time-consuming, and susceptible to human error (3–5). As the volume and complexity of biomedical literature continue to expand, these limitations increasingly constrain the efficiency and scalability of evidence synthesis. Recent advances in natural language processing have led to the development of large language models (LLMs) that can process and generate complex text (6–8), offering new opportunities to support and streamline systematic review processes (9–11).

Emerging evidence suggests that LLMs may assist data extraction tasks as a second reviewer, improving both efficiency and consistency when extracting information from structured and semi-structured research articles (12–14). Although these proof-of-concept studies have reported encouraging results and the potential to substantially reduce manual workload and human error, most evaluations have been limited to single models, small or restricted datasets, or single extraction runs (15–20). Moreover, several studies have relied on LLMs to assess their own outputs (12, 14, 21–24), raising concerns about reliability and bias. As a result, the accuracy and reproducibility of LLM-based data extraction across different models, prompt strategies, and repeated sessions under independent human adjudication remains insufficiently characterized.

To address these gaps, we conducted a prospective evaluation of three commercially available LLMs for data extraction in systematic reviews. Using five clinical questions (CQs) from the Japanese Clinical Practice Guidelines for the Management of Sepsis and Septic Shock 2024 (J-SSCG 2024) (25), we systematically assessed extraction accuracy, inter-session consistency, error characteristics, and processing efficiency of LLM-assisted data extraction for systematic reviews across different models and prompt engineering strategies.

## Methods

### Study design and settings

We conducted a prospective study to investigate the accuracy, reproducibility, and efficiency of LLM-assisted data extraction for systematic reviews. The study protocol was preregistered with the University Hospital Medical Information Network (UMIN000054461) and posted on the medRxiv preprint server to ensure transparency. Data extraction was performed between 23 May and 7 June 2024, followed by human review and adjudication conducted between 13 June 2024 and 16 January 2026.

### Clinical questions and literature selection

The J-SSCG 2024 was developed collaboratively by the Japanese Society of Intensive Care Medicine and the Japanese Association for Acute Medicine, building on the J-SSCG 2020 and addressing clinical scenarios specific to the Japanese healthcare context (25, 26). We applied the evaluation framework to five CQs from the J-SSCG 2024, as previously described (10) (Supplementary Table S1). Briefly, these CQs addressed the following topics: (1) balanced crystalloids vs. normal saline for fluid resuscitation; (2) higher vs. lower mean arterial pressure targets; (3) sodium bicarbonate therapy for severe metabolic acidosis; (4) hemodynamic resuscitation guided by lactate clearance, capillary refill time or mixed venous oxygen saturation; and (5) restrictive vs. liberal fluid management. For each CQ, comprehensive literature searches were conducted in PubMed, CENTRAL, and Ichushi-Web. Following title/abstract and full-text screening by guideline working group members, the final set of eligible studies was selected for the qualitative analysis (Supplementary Table S2). Background characteristics and outcome data were independently extracted by working group members. Both Japanese and English publications were included.

### Large language model-assisted data extraction

We evaluated three LLMs for data extraction in systematic reviews: ChatGPT-4o (OpenAI, San Francisco, CA, USA), released on May 13, 2024, Claude 3 Sonnet (Anthropic, San Francisco, CA, USA), released on March 14, 2024, and Gemini 1.5 Pro (Google DeepMind, Mountain View, CA, USA), released on May 23, 2024. All selected LLMs are capable of processing portable document format (PDF) files and generating outputs in response to user instructions. PDF files corresponding to the final set of studies included in the qualitative analysis were manually uploaded to each LLM through their respective web-based user interfaces. After manual initiation, automated data extraction was performed using predefined prompt instructions. Model versions and generation parameters (e.g., temperature) were not explicitly controlled. Prior to data extraction, PDF files that were not machine-readable were converted into searchable text formats using the optical character recognition (OCR) of Adobe Acrobat (Adobe Inc., San Jose, CA, USA). OCR-processed files were visually checked to confirm that the text was readable before data extraction. PDF files unsuitable for OCR because of low-quality scans were excluded from the evaluation. In addition, we either obtained confirmation from the model developers or verified publicly available privacy policies to ensure that user-provided inputs were not incorporated into model training data. The studies were processed using a predefined workflow, and the order of extraction was not randomized. To examine the reliability of LLM-assisted data extraction, the entire extraction procedure was repeated twice, at intervals of two to six weeks following the first extraction session. The time required to complete each data extraction session was also recorded to assess processing efficiency.

### Prompt engineering strategies

We conducted data extraction separately for two components: background characteristics (e.g., study design, eligibility criteria, participant demographics, and interventions) and clinical outcomes (e.g., mortality) (Supplementary Table S3). Detailed prompts used for background and outcome extraction are provided in the Supplementary Appendix. In addition to the original prompt, we tested the performance of modified prompts using the following two prompt engineering strategies:

Chain-of-thought (CoT): the original prompt preceded by the instruction phrase “Let's think step by step”, encouraging the model to reason through extraction tasks (Supplementary Appendix) (27).

Self-reflection (SR): the original prompt was followed by “Review your previous answer and find problems with your answer. Based on the problems you found, improve your answer”, prompting the model to critique and refine its output (Supplementary Appendix) (28).

### Accuracy assessment and error classification

In this study, the accuracy of extracted data was evaluated using a reference standard defined as data extracted using conventional systematic review methods by two or more human reviewers. When discrepancies, omissions, or inaccuracies were identified in the human-extracted data during the evaluation process, the reference standard was updated by re-examining the original publications in accordance with guidance from the Agency for Healthcare Research and Quality (29). The finalized background characteristics and outcome data served as the reference standard for assessing accuracy. The outputs generated by the LLMs were independently reviewed by two evaluators (T. O. and K. M.). In cases of disagreement, a third reviewer resolved the conflict.

Errors were classified using predefined criteria as major, minor, or no error, based on the accuracy and completeness of the extracted background information, as well as the potential impact of errors in outcome data on meta-analytic results (Table 1) (12). In addition, errors were further categorized into four types: missing data, incorrect data, fabricated data, and other errors (Table 2). Because background information consisted primarily of descriptive content, the evaluation focused on overall consistency with the reference standard rather than exact agreement. Given that no standardized framework currently exists for categorizing extraction errors in LLM-assisted systematic review workflows, these thresholds were defined pragmatically to distinguish minor deviations from substantial extraction errors.

**Table 1:** Assessment of accuracy in data extraction.

Accuracy	Background information	Outcome
Major errors	Significant issues which can influence the credibility or results of systematic review and meta-analyses, such as more than half of the extracted information is missed, or any critical information is incorrect, missed, or fabricated.	Issues that can change the results of SR or meta-analyses, potentially leading to incorrect conclusions, including an incorrect number of randomized patients, an incorrect number of patients assigned to intervention and control groups, an incorrect number of events in outcome measures, or incorrect items.
Minor errors	Approximately 60% or more of the information is correctly extracted based on the reference standard, but some minor issues are present, such as some portions of the information (20%–40%) is not extracted even though it is shown in the paper, or is inaccurately extracted. However, errors are unlikely to influence the SR and meta-analyses.	Minor issues that are unlikely to change the results of SR or meta-analyses or lead to incorrect conclusions such as errors in rounding.
No errors	Approximately 80% or more of the information is correctly and comprehensively extracted based on the reference standard.	No issues with the numbers.

SR, systematic review.

**Table 2:** Type of errors in data extraction.

Error type	Definitions
Missing data	Original information is present in the file but not extracted.
Incorrect data	Data extracted incorrectly from the source article, including the wrong population, outcome, time point, treatment group, or analysis set.
Fabricated data	False data considered to be hallucinations by an LLM.
Others	Not covered by the above definitions.

LLM, large language model.

### Statistical analysis

In the primary analysis, confusion matrices were constructed for each model, prompt, session, separately for two categories (background and outcome information). Accuracy was evaluated at the individual data-element (cell) level and defined as the proportion of data elements with no error. All data elements were weighted equally in the primary analysis. Then, error patterns were systematically summarized and described to explore potential mechanisms underlying extraction errors. Additionally, to evaluate reliability, outputs were compared across the three extraction sessions. Consistency was calculated using the following formula: Consistency rate (%) = number of concordant outputs/total number of outputs × 100. Furthermore, we calculated inter-reviewer agreement to assess the reliability of the evaluation process between the two reviewers. Inter-session consistency reflects reproducibility of LLM outputs, whereas inter-reviewer agreement reflects concordance between human evaluators. Finally, the time required for LLM-based data extraction was compared across different models and prompt engineering strategies.

As LLMs potentially used open access papers as training data, data contamination may be a limitation of this study. Therefore, we conducted a sensitivity analysis restricted to open access papers and non-open access papers and re-evaluated the performance of LLMs. All analyses except for the sensitivity analysis were predefined in the study protocol prior to data extraction.

Continuous variables were summarized as means with 95% confidence intervals (CIs). Categorical variables were reported as counts and percentages. All statistical analyses were conducted using GraphPad Prism version 10 (GraphPad Software, San Diego, CA, USA).

## Results

### Characteristics of included studies

Across the five CQs, 36 studies met inclusion criteria (Supplementary Table S2). Each session therefore involved extraction of 504 background data cells (14 items per study) and 208 outcome data cells (number of items depending on the specific CQ).

### Accuracy of data extraction

**Figure 1:**
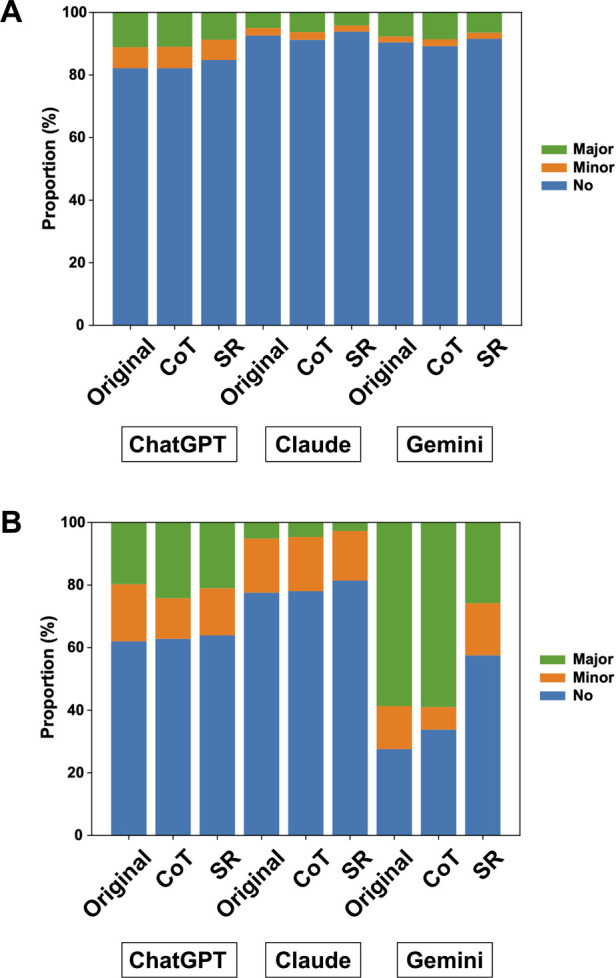
Proportion of no, minor, and major errors in data extraction across large language models and prompt strategies. Each panel shows the averaged proportion of no, minor, and major errors across sessions for extraction of background **(A)** and outcome variables **(B)** by each model (ChatGPT-4o, Claude 3 Sonnet, Gemini 1.5 Pro) using the original, chain-of-thought (CoT), and self-reflection (SR) prompts.

For background characteristics, the mean no-error proportion was 81.6% (95% CI, 76.3–87.0) for ChatGPT-4o, 92.4% (95% CI, 87.6–97.2) for Claude 3 Sonnet, and 90.2% (95% CI, 87.1–93.2) for Gemini 1.5 Pro, averaged across the three sessions. Prompt engineering strategies resulted in only modest changes in background extraction accuracy across all models (ChatGPT-4o CoT: 82.2% [95% CI, 77.3–87.2]; ChatGPT-4o SR: 85.0% [95% CI, 82.0–88.1]; Claude 3 CoT: 91.0% [95% CI, 87.3–94.7]; Claude 3 SR: 93.5% [95% CI, 91.9–95.1]; Gemini 1.5 Pro CoT: 89.2% [95% CI, 86.9–91.4]; Gemini 1.5 Pro SR: 91.7% [95% CI, 89.0–94.3]) (Figure 1, Supplementary Figures S1–S4, and Supplementary Tables S4–S6).

For outcome data extraction, the mean no-error proportion was 64.4% (95% CI, 52.3–76.6) for ChatGPT-4o, 80.7% (95% CI, 65.6–95.9) for Claude 3 Sonnet, and 27.8% (95% CI, 3.8–51.7) for Gemini 1.5 Pro, averaged across sessions. While ChatGPT-4o and Claude 3 Sonnet showed comparable accuracy, substantial variability was observed across prompt strategies in Gemini 1.5 Pro, especially with the SR prompt [63.0% (95% CI, 41.3–84.6)].

### Types of errors

Error-type analyses (Table 3 and Supplementary Tables S7, S8) revealed that missing or incorrect data accounted for the majority of errors [91.4% (95% CI, 88.4–94.5)] across all models. For background and outcome data extraction, the frequency of missing data was 67.3% (95% CI, 52.5–82.1) and 83.4% (95% CI, 74.7–92.2) for ChatGPT-4o, 57.7% (95% CI, 42.7–72.6) and 73.3% (95% CI, 31.0–100.0) for Claude 3 Sonnet, and 39.5% (95% CI, 0.0–86.4) and 92.6% (95% CI, 80.4–100.0) for Gemini 1.5 Pro. The frequencies for fabricated outputs for background and outcome data extraction were 2.7% (95% CI, 0.0–6.0) and 4.5% (95% CI, 0.0–9.8) for ChatGPT-4o, 0.7% (95% CI, 0.0–2.8) and 10.0% (95% CI, 0.0–37.8) for Claude 3 Sonnet, and 14.4% (95% CI, 0.0–38.1) and 3.9% (95% CI, 0.0–13.4) for Gemini 1.5 Pro. Prompt engineering strategies had minimal impact on the overall frequency of missing data, with the exception of the SR prompt for Gemini 1.5 Pro in background data extraction [8.2% (95% CI, 0.0–26.8)].

**Table 3:** Type of errors in data extraction in the first session.

Category	LLM	Prompt strategy	Type of error			
			Missing	Incorrect	Fabricated	Others
Background	ChatGPT-4o	Original	46 (58.2%)	21 (26.6%)	2 (2.5%)	10 (12.7%)
		Chain-of-thought	60 (64.5%)	22 (23.7%)	3 (3.2%)	8 (8.6%)
		Self-reflection	61 (65.6%)	19 (20.4%)	3 (3.2%)	10 (10.8%)
	Claude 3 Sonnet	Original	15 (45.5%)	17 (51.5%)	1 (3.0%)	0 (0.0%)
		Chain-of-thought	22 (52.4%)	17 (40.5%)	1 (2.4%)	2 (4.8%)
		Self-reflection	10 (37.0%)	15 (55.6%)	1 (3.7%)	1 (3.7%)
	Gemini 1.5 Pro	Original	13 (25.0%)	26 (50.0%)	11 (21.2%)	2 (3.8%)
		Chain-of-thought	25 (47.2%)	24 (45.3%)	4 (7.5%)	0 (0.0%)
		Self-reflection	15 (40.5%)	21 (56.8%)	1 (2.7%)	0 (0.0%)
Outcome	ChatGPT-4o	Original	62 (80.5%)	11 (14.3%)	2 (2.6%)	2 (2.6%)
		Chain-of-thought	63 (78.8%)	11 (13.8%)	5 (6.3%)	1 (1.3%)
		Self-reflection	77 (84.6%)	10 (11.0%)	2 (2.2%)	2 (2.2%)
	Claude 3 Sonnet	Original	26 (83.9%)	1 (3.2%)	2 (6.5%)	2 (6.5%)
		Chain-of-thought	31 (81.6%)	2 (5.3%)	3 (7.9%)	2 (5.3%)
		Self-reflection	41 (87.2%)	2 (4.3%)	2 (4.3%)	2 (4.3%)
	Gemini 1.5 Pro	Original	136 (95.8%)	5 (3.5%)	1 (0.7%)	1 (0.7%)
		Chain-of-thought	126 (92.6%)	3 (2.2%)	5 (3.7%)	2 (1.5%)
		Self-reflection	73 (91.3%)	5 (6.3%)	0 (0.0%)	2 (2.5%)

LLM, large language model.

### Consistency across sessions

**Figure 2:**
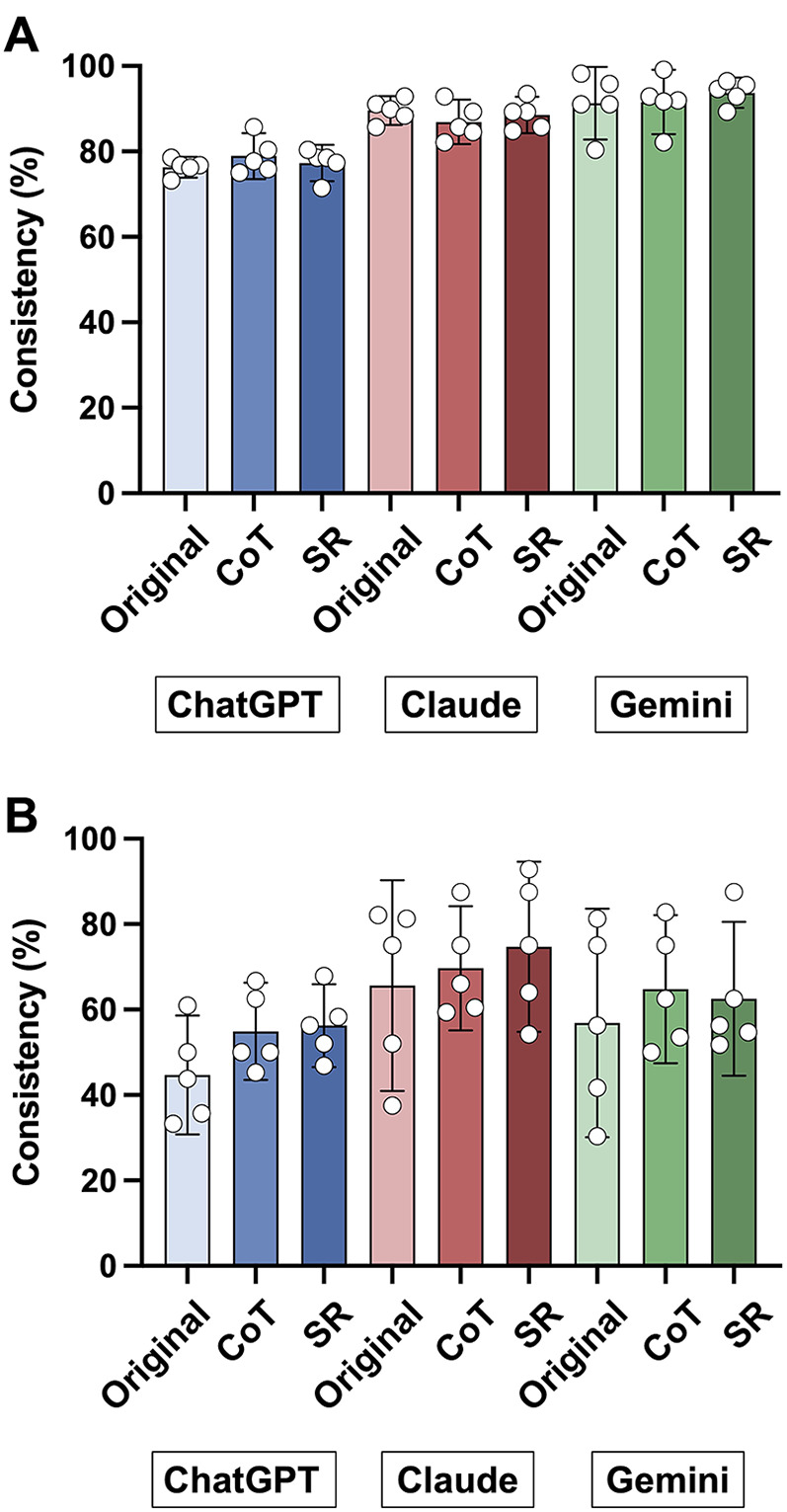
Inter-session consistency across large language models and prompt strategies. Each panel shows the proportion of consistent outputs for extracting background **(A)** and outcome variables **(B)** by each model (ChatGPT-4o, Claude 3 Sonnet, Gemini 1.5 Pro) using the original, chain-of-thought (CoT), and self-reflection (SR) prompts. Consistency rate (%) was calculated as the number of concordant outputs divided by the total number of outputs, multiplied by 100. Open circles represent individual consistency rates for each clinical question.

The mean consistency rate for background data extraction averaged was 76.3% (95% CI, 73.9–78.7) for ChatGPT-4o, 89.6% (95% CI, 86.2–93.0) for Claude 3 Sonnet, and 91.3% (95% CI, 82.8–99.8) for Gemini 1.5 Pro (Figure 2A). For outcome data extraction (Figure 2B), the mean consistency rate averaged across CQs was 44.8% (95% CI, 30.8–58.7) for ChatGPT-4o, 65.6% (95% CI, 40.9–90.3) for Claude 3 Sonnet, and 56.9% (95% CI, 30.2–83.7) for Gemini 1.5 Pro.

### Inter-rater agreement between two reviewers

**Figure 3:**
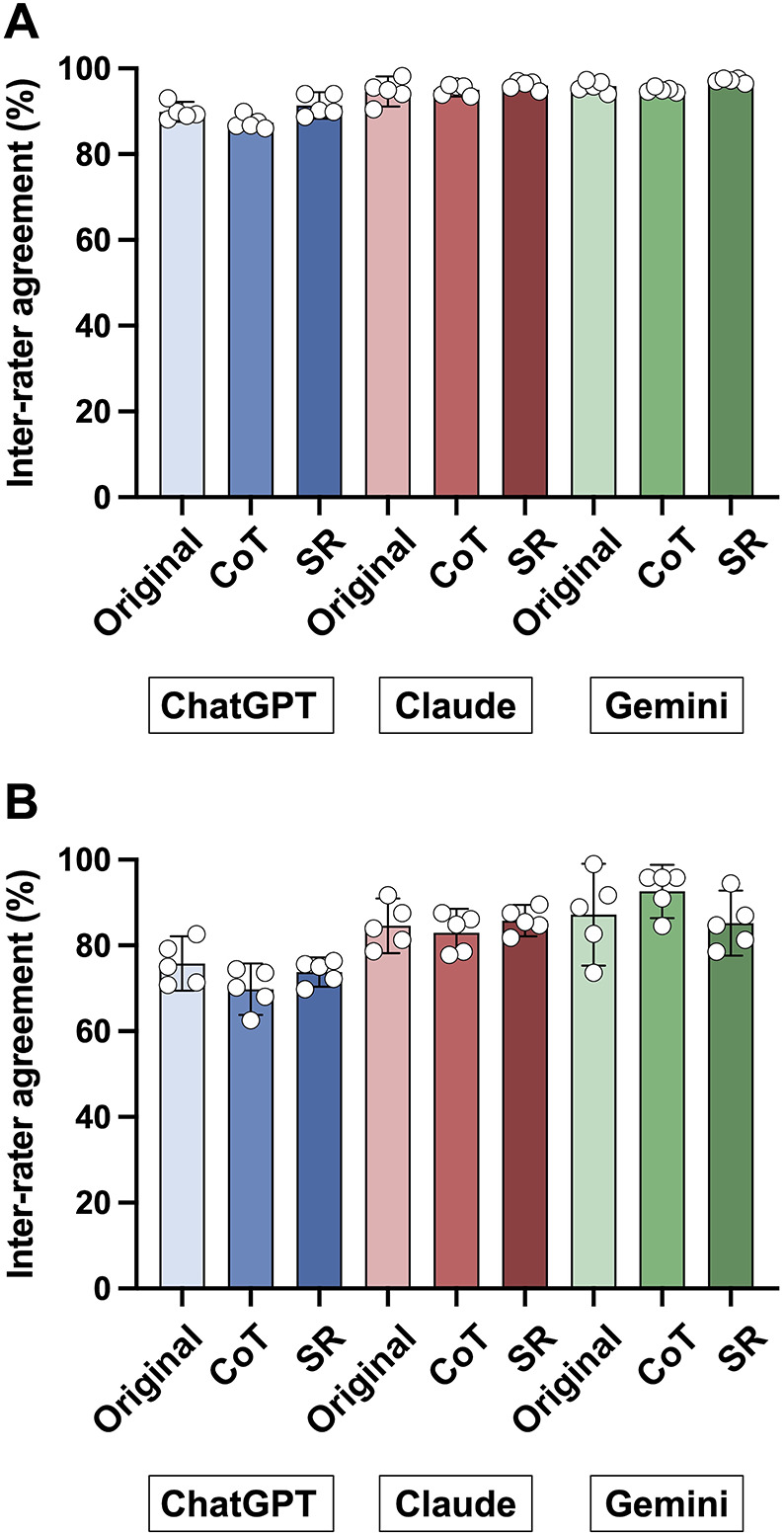
Inter-rater agreement across large language models and prompt strategies. Inter-reviewer agreement (%) between two human reviewers, averaged across sessions, is expressed as the percentage of extraction cells for which both reviewers classified the model output identically (error/non-error). Open circles represent individual agreement rates for each clinical question. Each panel shows the agreement rate for extracting background **(A)** and outcome variables **(B)**. CoT: chain-of-thought; SR: self-reflection.

For background data, inter-rater agreement was 89.9% (95% CI, 87.5–92.2) for ChatGPT-4o, 94.6% (95% CI, 91.2–98.1) for Claude 3 Sonnet, and 95.9% (95% CI, 94.3–95.7) for Gemini 1.5 Pro. Inter-rater agreement for outcome data extraction was 75.8% (95% CI, 69.4–82.1) for ChatGPT-4o, 84.6% (95% CI, 78.2–91.0) for Claude 3 Sonnet, and 87.2% (95% CI, 75.3–99.0) for Gemini 1.5 Pro, with variability among CQs and prompt engineering strategies (Figure 3 and Supplementary Figures S6–S8).

### Processing time for data extraction

**Figure 4:**
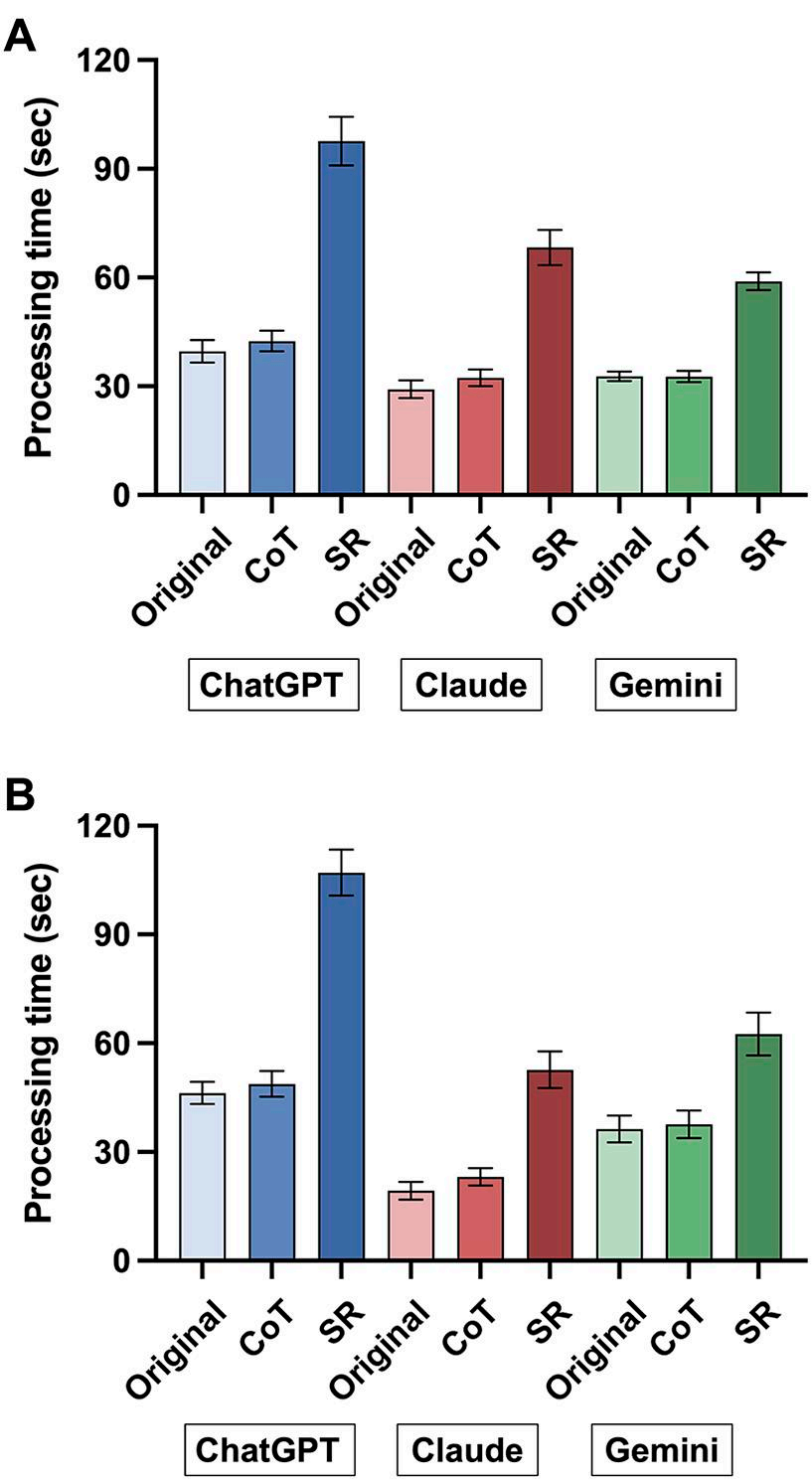
Processing time across large language models and prompt strategies. Each panel shows the processing time averaged across sessions for extracting background **(A)** and outcome variables **(B)** per article by each model (ChatGPT-4o, Claude 3 Sonnet, Gemini 1.5 Pro) using the original, chain-of-thought (CoT), and self-reflection (SR) prompts.

Processing time per article varied across models and prompt strategies for both background and outcome data extraction (Figures 4A,B). The mean processing time for background data extraction was 39.7 s (95% CI, 36.6–42.8) for ChatGPT-4o, 29.2 s (95% CI, 26.7–31.6) for Claude 3 Sonnet, and 32.7 s (95% CI, 31.4–34.0) for Gemini 1.5 Pro. For outcome data extraction, the mean processing time was 46.3 s (95% CI, 43.2–49.4) for ChatGPT-4o, 19.3 s (95% CI, 16.9–21.8) for Claude 3 Sonnet, and 36.3 s (95% CI, 32.7–40.0) for Gemini 1.5 Pro. When SR prompts were used, the mean processing time for background data extraction increased to 97.7 s (95% CI, 91.0–104.4) for ChatGPT-4o, 68.3 s (95% CI, 63.5–73.2) for Claude 3 Sonnet, and 59.0 s (95% CI, 56.5–61.4) for Gemini 1.5 Pro. For outcome data extraction, the corresponding processing times were 107.1 s (95% CI, 100.7–113.5), 52.7 s (95% CI, 47.7–57.7), and 62.5 s (95% CI, 56.7–68.4), respectively.

### Sensitivity analysis

For background data extraction, the mean no-error proportion for open-access and paywalled articles, respectively, was 80.9% (95% CI, 70.5–91.4) and 82.7% (95% CI, 78.1–87.2) for ChatGPT-4o, 94.1% (95% CI, 90.3–97.9) and 92.2% (95% CI, 87.8–96.5) for Claude 3 Sonnet, and 91.4% (95% CI, 88.3–94.6) and 88.7% (95% CI, 82.0–95.4) for Gemini 1.5 Pro. For outcome data extraction, the mean no-error proportion for open-access and paywalled articles, respectively, was 60.5% (95% CI, 52.4–68.5) and 67.2% (95% CI, 47.7–86.6) for ChatGPT-4o, 78.6% (95% CI, 63.1–94.1) and 83.6% (95% CI, 61.4–105.8) for Claude 3 Sonnet, and 32.9% (95% CI, 0.0–76.0) and 20.1% (95% CI, 0.0–44.1) for Gemini 1.5 Pro (Supplementary Figure S9).

## Discussion

### Principal findings

In this prospective, multi-session evaluation, we found that commercially available LLMs can reliably support background data extraction for systematic reviews, whereas outcome data extraction remains substantially more variable across models and sessions than background data extraction. Accuracy differed among models, with mean no-error proportions ranging from 82% to 92% for background data extraction and from approximately 28% to 81% for outcome data extraction. Prompt engineering strategies, particularly the SR prompt, provided modest improvements but did not overcome model-specific limitations. These findings indicate that model choice and prompt design may both affect the performance of data extraction. Human oversight therefore remains essential, particularly for outcome-level data extraction.

### Comparison with prior work

A key observation of this study is the clear distinction between background and outcome data extraction. Consistent with prior reports, background characteristics were extracted with high accuracy by recent LLMs (12, 14–16), likely reflecting the standardized and explicitly reported nature of these items. In contrast, outcome data extraction, which often involves complex statistics and more nuanced contextual information (17, 22, 24), was substantially more challenging. By systematically evaluating outcome-level performance across multiple sessions, our study demonstrated that this limitation is persistent and model-dependent, highlighting an important gap in the current applicability of LLMs for fully automated systematic reviews.

Previous studies have reported high performance by leading LLMs for structured information extraction tasks. For example, one study demonstrated that GPT-4 achieved precision and recall approaching 90% for automated extraction of structured scientific data using prompt-engineered workflows (18). Similarly, another study that compared Claude 2 and GPT-4 for extracting predefined data elements from systematic review articles reported high extraction accuracy for both models, with Claude 2 achieving an accuracy of approximately 96% (20). Consistent with these findings, our results also suggest that extraction performance varies across models, indicating that extraction limitations are not solely task-dependent but also model-dependent. These differences may reflect variations in model architecture, training data sources, or alignment strategies, which may influence the ability of LLMs to accurately extract complex numerical information.

Our findings further indicated that not only model selection but also prompt engineering strategies influence performance, emphasizing prompt optimization as a primary means of improving LLM outputs (18, 19). Several recent methodological studies have demonstrated that structured prompt design and formal evaluation frameworks can improve LLM performance in systematic review-related tasks. A previous study reported improvement in PICO-based query generation using tailored prompting strategies across different LLMs, highlighting the importance of task-specific prompt design (30). Another study evaluated the performance of LLM-generated structured abstracts using the PRISMA-A framework, demonstrating that checklist-based evaluation and structured prompting can enhance output quality (31). In our study, CoT and SR prompts yielded modest improvements in performance in selected scenarios, particularly for outcome extraction with Claude 3 Sonnet and Gemini 1.5 Pro, but these strategies did not overcome intrinsic model limitations. These findings suggest that prompt engineering may play a larger role in the extraction of complex numerical outcome data, whereas overall performance appears to remain more closely related to underlying model capabilities.

In addition to prompt engineering strategies, several studies have explored alternative approaches that combine LLMs with custom pipelines or task-specific applications. For example, a recent study showed that providing structured templates or examples of correctly formatted output can improve extraction accuracy in automated systematic review systems (17). These observations highlight the potential of integrating LLMs with task-specific architectures, although they often require additional engineering effort beyond simple prompt design. In contrast, our study focused on evaluating the performance of commercially available LLMs under standardized prompting strategies, thereby providing a clearer assessment of their baseline capabilities for automated data extraction.

Error-type analyses provided additional insight into the mechanisms underlying incorrect LLM outputs during data extraction. Earlier evaluations have primarily focused on aggregate accuracy metrics without detailed characterization of error types (14, 21). Although several recent studies have attempted to classify errors using quantitative and qualitative approaches (12, 20, 24), detailed comparisons across models and outcome complexity remain limited. In our analysis, errors generated by LLMs during data extraction were predominantly missing or incorrect values, whereas fabricated outputs were relatively uncommon. These findings are notable because hallucination is often considered a major limitation of LLMs in scientific applications. Our results instead suggest that most errors arise from incomplete identification or incorrect extraction of numerical values rather than the generation of entirely fabricated information. Several factors may contribute to these patterns. Outcome data in clinical studies are often distributed across multiple sections, tables, or supplementary material and may involve complex reporting structures such as multiple time points, subgroup analyses, or numerator–denominator formats. These features may increase the likelihood that models overlook relevant information or misidentify numerical relationships during extraction. Collectively, these findings emphasize the importance of evaluating error types rather than relying solely on summary accuracy metrics when assessing LLM performance in systematic review workflows.

Reproducibility across sessions represents another key contribution of this study. Prior investigations have typically evaluated LLM performance in single-run settings (14, 22), implicitly assuming reproducibility. However, because LLMs generate responses through probabilistic token sampling, identical prompts do not necessarily yield identical outputs. Our repeated-session design revealed that inter-session consistency was high for background data but declined markedly for outcome data, even when identical prompts were used. As a plausible explanation, outcome data extraction often involves identifying numerical values from complex reporting structures, including multiple time points, subgroup analyses, or complex table formats, which may increase ambiguity and contribute to variability across runs. This finding has important implications for systematic review workflows, as inconsistent outputs may require additional verification steps when LLMs are used for automated extraction.

Claude 3 Sonnet demonstrated the most consistent performance, followed by ChatGPT-4o, whereas Gemini 1.5 Pro showed greater variability. Although the internal mechanisms underlying these differences cannot be fully explained due to the black-box nature of LLM architectures, the greater stability of Claude 3 Sonnet may reflect stronger instruction-following behavior or more conservative extraction strategies when interpreting numerical information. These findings suggest that reproducibility should be considered a core evaluation domain for LLM-assisted systematic reviews, particularly when extracting outcome data.

Earlier reports have shown that LLMs can markedly reduce data extraction time compared with manual approaches (17, 21, 24). In this study, we further demonstrated that gains in accuracy achieved through more complex prompt strategies, such as the SR prompt, are accompanied by increased processing time. Although this trade-off has received limited attention in prior studies, a moderate increase in processing time may be acceptable in many research settings if it results in improved extraction accuracy. In large-scale systematic reviews or guideline development projects, researchers may reasonably tolerate processing times of several minutes per article when accuracy gains reduce the need for manual verification and correction. One possible explanation for the longer processing time observed with the SR prompt is that more structured prompting may encourage the model to perform additional reasoning steps or internal consistency checks before generating the final output. In this context, longer processing times may reflect more deliberate reasoning rather than inefficiency. These findings highlight the importance of considering both accuracy and computational time when designing LLM-assisted systematic review workflows.

Our sensitivity analysis showed comparable performance for open-access and paywalled articles. Although previous proof-of-concept studies have relied primarily on open-access samples (12), our findings suggest that extraction performance cannot be explained solely by prior exposure to openly available training data.

### Implications

LLMs show promise for expediting data extraction in systematic reviews, particularly for retrieving structured background information. When combined with prompt engineering strategies and human oversight, LLMs could reduce workload and accelerate guideline development. However, our results caution against fully automated extraction of clinical outcomes. Even the best model made errors one in five outcome cells (mostly missing or incorrect data), and hallucinations were observed, though at lower rates. Human reviewers therefore remain essential for verifying data. In settings where time or resources are limited, LLMs could prioritize extraction tasks or serve as an initial reviewer, with humans resolving ambiguous cases.

### Limitations

This study has several limitations. First, the evaluation was restricted to trials addressing sepsis and was based on studies derived from a single clinical guideline context. The analysis included a limited number of studies, and generalizability to other clinical domains remains uncertain. Because the complexity of data extraction may vary across medical specialties and study designs, the present findings may not be directly applicable to other areas of systematic review. Second, the models were evaluated based on data extraction performed between May 23 and June 7, 2024; multiple LLM updates have been released since, and accuracy may have improved. Because LLM performance may change substantially with version updates, our findings should be interpreted in the context of the specific model versions evaluated. Third, we did not control model versions and generation parameters, including temperature, because models were accessed through web-based user interfaces. Fourth, we did not assess the impact of cell-level accuracy on pooled estimates. Because missing values and inconsistencies in extracted variables often prevented construction of comparable meta-analytic datasets, we considered that a full meta-analytic sensitivity analysis based solely on model-extracted data was not feasible. Finally, our evaluation relied on human assessment as the reference standard; however, human reviewers are not infallible, and the gold standard itself may contain errors. Although human judgement is generally considered more reliable than automated extraction for identifying hallucinations and ensuring validity, inter-rater variability and oversight may have affected the accuracy of our reference data. Future work should explore hybrid pipelines in which LLMs assist human reviewers rather than replace them, potentially improving both the efficiency and reliability of the evaluation process.

In conclusion, commercially available LLMs can effectively support background data extraction in systematic reviews, with only modest improvements achieved through prompt engineering. However, outcome data extraction remains challenging, emphasizing the continued need for human review. Careful model selection and prompt engineering strategies are critical for the responsible integration of LLMs into evidence synthesis workflows.

## Data Availability

The raw data supporting the conclusions of this article will be made available by the authors, without undue reservation.
