# Supplementary material for: Performance of large language models and prompt engineering strategies for data extraction in systematic reviews
Source: Front Digit Health. 2026 Apr 29;8:1799623. doi: 10.3389/fdgth.2026.1799623 (PMC13168097; doi:10.3389/fdgth.2026.1799623)
Supplement: Supplementary file 1 [file Supplementaryfile1.docx]

**Supplementary Material**

**Performance of large language models and prompt engineering strategies in data extraction for systematic reviews**

**Authors:** Takehiko Oami, M.D., Ph.D.^1^, Yohei Okada, M.D., Ph.D.^2^, Kenjiro Maeda, M.D.^1^, Taka-aki Nakada, M.D., Ph.D.^1^

**Affiliations:**

1. Department of Emergency and Critical Care Medicine, Chiba University Graduate School of Medicine, Chiba, Japan

2. Department of Preventive Services, Kyoto University Graduate School of Medicine, Kyoto, Japan

**Supplementary Appendix.** Supplementary appendix

**Supplementary Table S1.** List of the patient/population/problem, intervention, comparison, and outcomes of the selected clinical questions

**Supplementary Table S2.** List of included studies for data extraction

**Supplementary Table S3.** List of outcome variables for data extraction

**Supplementary Table S4.** Number of no, minor, and major errors in data extraction across large language models and prompt strategies in the first session

**Supplementary Table S5.** Number of no, minor, and major errors in data extraction across large language models and prompt strategies in the second session

**Supplementary Table S6.** Number of no, minor, and major errors in data extraction across large language models and prompt strategies in the third session

**Supplementary Table S7.** Types of errors in data extraction in the second session

**Supplementary Table S8.** Types of errors in data extraction in the third session

**Supplementary Figure S1.** Proportion of no, minor, and major errors in data extraction across large language models and prompt strategies in the first session

**Supplementary Figure S2.** Proportion of no, minor, and major errors in data extraction across large language models and prompt strategies in the second session

**Supplementary Figure S3.** Proportion of no, minor, and major errors in data extraction across large language models and prompt strategies in the third session

**Supplementary Figure S4.** Proportion of accuracy across large language models and prompt strategies

**Supplementary Figure S5.** Distribution of major and minor errors in background data extraction across large language models and prompt strategies

**Supplementary Figure S6.** Inter-rater agreement across large language models and prompt strategies in the first session

**Supplementary Figure S7.** Inter-rater agreement across large language models and prompt strategies in the second session

**Supplementary Figure S8.** Inter-rater agreement across large language models and prompt strategies in the third session

**Supplementary Figure S9.** Comparison of accuracy in data extraction in large language model-based data extraction between open access and non-open access publications

**Supplementary Appendix**

**Table of Contents**

**1. Original prompt for data extraction**

**2. Modified prompt with the chain-of-thought strategy for data extraction**

**3. Modified prompt with the self-reflection strategy for data extraction**

**Supplementary Appendix**

**1. Original prompt for data extraction**

We used the following prompt to extract the background information.

“You are conducting a systematic review and meta-analysis, focusing on a specific area of medical research. Your task is to read through the imported PDF file and to extract background information about study and participants. Please summarize the extracted information and export it to an Excel file. If there is no relevant information in the PDF file, please enter “not available” in the cell. If the word count of the extracted data for each information is over 300 words, please summarize the descriptions and indicate that the content is being summarized. The required information is as follows:

# Title of the article

# Name of first author

# Year of publication

# Journal name

# Study design

# Inclusion criteria

# Exclusion criteria

# Number of participants to be randomized

# Number of participants enrolled in the intervention and control groups

# Average age of study participants in the intervention and control groups

# Sex of participants in the intervention and control groups

# Severity scores or scales such as Sequential Organ Failure Assessment (SOFA) and Acute Physiology and Chronic Health Evaluation (APACHE) in the intervention group and the control groups

# Details of interventions

# Details of controls

We used the following prompt to extract outcome information:

We will use the following prompt to extract the background information.

“You are conducting a systematic review and meta-analysis, focusing on a specific area of medical research. Your task is to read through the imported PDF file and to extract outcome information about the participants. In extracting the outcomes, outcomes based on the intention-to-treat (ITT) analysis should be prioritized if available. If there is no outcome data based on the ITT analysis, please extract outcomes based on the per protocol analysis. If there is no clear description of the type of analysis written, please use the results from the main analysis. In such cases, please add the type of analysis, including ITT, per protocol, or unknown to the extracted data. Please summarize the extracted information and export it to an Excel file.

If the outcome is a dichotomous variable, please extract the number of events and participants in each group. If the outcome is a continuous variable, please extract the mean and standard deviation with the number of participants. If the outcome is provided with median and interquartile range (IQR) or mean and 95% confidence interval, please extract them as they are and add the description what the number is. If actual values are not provided in the text or table, please estimate the outcome value from figures or tables in the PDF file. Please add the type of continuous variables according to the extracted subjects. The list of outcome variables is as follows:

# Outcome variable 1

# Outcome variable 2

# Outcome variable 3

# Outcome variable 4”

**2. Modified prompt with the chain-of-thought strategy for data extraction**

We used the following prompt for background information.

“You are conducting a systematic review and meta-analysis, focusing on a specific area of medical research. Your task is to read through the imported PDF file and to extract background information about study and participants. Please summarize the extracted information and export it to a Google spreadsheet. If there is no relevant information in the PDF file, please enter “not available” in the cell. If the word count of the extracted data for each information is over 300 words, please summarize the descriptions and indicate that the content is being summarized. The required information is as follows:

# Title of the article

# Name of first author

# Year of publication

# Journal name

# Study design

# Inclusion criteria

# Exclusion criteria

# Number of participants to be randomized

# Number of participants enrolled in the intervention and control groups

# Average age of study participants in the intervention and control groups

# Sex of participants in the intervention and control groups

# Severity scores or scales such as Sequential Organ Failure Assessment (SOFA) and Acute Physiology and Chronic Health Evaluation (APACHE) in the intervention group and the control groups

# Details of interventions

# Details of controls

Let’s think step-by-step.”

We used the following prompt for outcome information.

“You are conducting a systematic review and meta-analysis, focusing on a specific area of medical research. Your task is to read through the imported PDF file and to extract outcome information about the participants. In extracting the outcomes, outcomes based on the intention-to-treat (ITT) analysis should be prioritized if available. If there is no outcome data based on the ITT analysis, please extract outcomes based on the per protocol analysis. If there is no clear description of the type of analysis written, please use the results from the main analysis. In such cases, please add the type of analysis, including ITT, per protocol, or unknown to the extracted data. Please summarize the extracted information and export it to a Google spreadsheet.

If the outcome is a dichotomous variable, please extract the number of events and participants in each group. If the outcome is a continuous variable, please extract the mean and standard deviation with the number of participants. If the outcome is provided with median and interquartile range (IQR) or mean and 95% confidence interval, please extract them as they are and add the description what the number is. If actual values are not provided in the text or table, please estimate the outcome value from figures or tables in the PDF file. Please add the type of continuous variables according to the extracted subjects. The list of outcome variables is as follows:

# 90-day mortality

# Short-term mortality

# Severe acute kidney injury or renal replacement therapy

# Ventilator-free days

# Severe adverse events

# Vasopressor-free days

# Lung edema

# Amount of fluid administration

Let’s think step-by-step.”

**3. Modified prompt with the self-reflection strategy for data extraction**

We used the following prompt for background information.

“You are conducting a systematic review and meta-analysis, focusing on a specific area of medical research. Your task is to read through the imported PDF file and to extract background information about study and participants. Please summarize the extracted information and export it to a Google spreadsheet. If there is no relevant information in the PDF file, please enter “not available” in the cell. If the word count of the extracted data for each information is over 300 words, please summarize the descriptions and indicate that the content is being summarized. The required information is as follows:

# Title of the article

# Name of first author

# Year of publication

# Journal name

# Study design

# Inclusion criteria

# Exclusion criteria

# Number of participants to be randomized

# Number of participants enrolled in the intervention and control groups

# Average age of study participants in the intervention and control groups

# Sex of participants in the intervention and control groups

# Severity scores or scales such as Sequential Organ Failure Assessment (SOFA) and Acute Physiology and Chronic Health Evaluation (APACHE) in the intervention group and the control groups

# Details of interventions

# Details of controls

Review your previous answer and find problems with your answer. Based on the problems you found, improve your answer.”

We used the following prompt for outcome information.

“You are conducting a systematic review and meta-analysis, focusing on a specific area of medical research. Your task is to read through the imported PDF file and to extract outcome information about the participants. In extracting the outcomes, outcomes based on the intention-to-treat (ITT) analysis should be prioritized if available. If there is no outcome data based on the ITT analysis, please extract outcomes based on the per protocol analysis. If there is no clear description of the type of analysis written, please use the results from the main analysis. In such cases, please add the type of analysis, including ITT, per protocol, or unknown to the extracted data. Please summarize the extracted information and export it to a Google spreadsheet.

If the outcome is a dichotomous variable, please extract the number of events and participants in each group. If the outcome is a continuous variable, please extract the mean and standard deviation with the number of participants. If the outcome is provided with median and interquartile range (IQR) or mean and 95% confidence interval, please extract them as they are and add the description what the number is. If actual values are not provided in the text or table, please estimate the outcome value from figures or tables in the PDF file. Please add the type of continuous variables according to the extracted subjects. The list of outcome variables is as follows:

# 90-day mortality

# Short-term mortality

# Severe acute kidney injury or renal replacement therapy

# Ventilator-free days

# Severe adverse events

# Vasopressor-free days

# Lung edema

# Amount of fluid administration

Review your previous answer and find problems with your answer. Based on the problems you found, improve your answer.”

**Supplementary Table S1. List of the patient/population/problem, intervention, and comparison of the selected clinical questions**

| CQ | Patient, population, problem | Intervention | Comparison |
| --- | --- | --- | --- |
| CQ1 | Adult patients (18 years old or older) diagnosed with or suspected of having infection, bacteremia, or sepsis | Balanced crystalloid administration | 0.9% sodium chloride administration |
| CQ2 | Adult patients (18 years old or older) with sepsis or suspected as sepsis, infection, bacteremia or patients admitted to ICU | Targeting a higher mean arterial pressure | Targeting a lower mean arterial pressure |
| CQ3 | Adult patients (18 years old or older) with sepsis presenting with severe metabolic acidosis or patients admitted to ICU | Sodium bicarbonate administration | No sodium bicarbonate administration |
| CQ4 | Adult patients (18 years old or older) with sepsis or septic shock | Usual care with at least one of the following tissue perfusion parameters: lactate/lactate clearance, capillary refill time, ScvO_2_/SvO_2_, and P(v-a) CO2/C (a-v) O_2_. | Usual care with different parameters mentioned in the interventional group or standard care without the utilization of any specific tissue perfusion parameters |
| CQ5 | Adult patients (18 years old or older) with sepsis, sepsis-induced hypotension, or septic shock | Restrictive fluid management aiming to reduce the amount of fluid therapy for up to 24 h | Conventional fluid management or non-restrictive fluid management defined by authors |

CQ: clinical question; ICU: intensive care unit

**Supplementary Table S2. List of included studies for data extraction**

| CQ | Year | Journal | Title | Open access |
| --- | --- | --- | --- | --- |
| CQ1 | 2022 | N Engl J Med | Balanced Multielectrolyte Solution versus Saline in Critically Ill Adults | No |
|  | 2022 | Hong Kong Journal of Emergency Medicine | 0.9% Saline v/s Ringer's Lactate for fluid resuscitation in adult sepsis patients in emergency medical services: An open label Randomized Controlled trial | Yes |
|  | 2017 | Am. J. Respir. Crit. Care Med. | Balanced Crystalloids versus Saline in the Intensive Care Unit. The SALT Randomized Trial | Yes |
|  | 2018 | N Engl J Med | Balanced Crystalloids versus Saline in Critically Ill Adults | No |
|  | 2019 | Am. J. Respir. Crit. Care Med. | Balanced Crystalloids versus Saline in Sepsis: A Secondary Analysis of the SMART Clinical Trial | Yes |
|  | 2016 | Critical care and resuscitation | A multicentre, randomised controlled pilot study of fluid resuscitation with saline or Plasma-Lyte 148 in critically ill patients | Yes |
|  | 2015 | JAMA | Effect of a Buffered Crystalloid Solution vs Saline on Acute Kidney Injury Among Patients in the Intensive Care Unit: the SPLIT Randomized Clinical Trial | Yes |
|  | 2021 | JAMA | Effect of Intravenous Fluid Treatment With a Balanced Solution vs 0.9% Saline Solution on Mortality in Critically Ill Patients: The BaSICS Randomized Clinical Trial | Yes |
| CQ2 | 2014 | New England Journal of Medicine | High versus low blood-pressure target in patients with septic shock | No |
|  | 2016 | Intensive Care Medicine | Higher versus lower blood pressure targets for vasopressor therapy in shock: a multicentre pilot randomized controlled trial | Yes |
|  | 2020 | JAMA | Effect of Reduced Exposure to Vasopressors on 90-Day Mortality in Older Critically Ill Patients With Vasodilatory Hypotension: A Randomized Clinical Trial | Yes |
|  | 2021 | Health Technology Assessment | Reduced exposure to vasopressors through permissive hypotension to reduce mortality in critically ill people aged 65 and over: the 65 RCT | Yes |
| CQ3 | 1990 | Ann Intern Med | Bicarbonate does not improve hemodynamics in critically ill patients who have lactic acidosis. A prospective, controlled clinical study | No |
|  | 2018 | Lancet | Sodium bicarbonate therapy for patients with severe metabolic acidaemia in the intensive care unit (BICAR-ICU): a multicentre, open-label, randomised controlled, phase 3 trial | No |
|  | 2023 | Crit Care Med | Long-Term Outcome of Severe Metabolic Acidemia in ICU Patients, a BICAR-ICU Trial Post Hoc Analysis | No |
|  | 1991 | Crit Care Med | Effects of bicarbonate therapy on hemodynamics and tissue oxygenation in patients with lactic acidosis: a prospective, controlled clinical study | No |
| CQ4 | 2014 | N Engl J Med | A randomized trial of protocol-based care for early septic shock | No |
|  | 1995 | NEJM | A TRIAL OF GOAL-ORIENTED HEMODYNAMIC THERAPY IN CRITICALLY ILL PATIENTS | No |
|  | 2001 | NEJM | EARLY GOAL-DIRECTED THERAPY IN THE TREATMENT OF SEVERE SEPSIS AND SEPTIC SHOCK | No |
|  | 2022 | Journal of Intensive Care Medicine | Early Lactate-Guided Resuscitation of Elderly Septic Patients | No |
|  | 2010 | Am J Respir Crit Care Med | Early Lactate-Guided therapy in Intensive care unit patients | Yes |
|  | 2019 | JAMA | Effect of a Resuscitation Strategy Targeting Peripheral Perfusion Status vs Serum Lactate Levels on 28-Day Mortality Among Patients With Septic Shock The ANDROMEDA-SHOCK Randomized Clinical Trial | Yes |
|  | 2020 | Ann Intensive Care | Effects of capillary refill time-vs. lactate-targeted fluid resuscitation on regional, microcirculatory and hypoxia-related perfusion parameters in septic shock: a randomized controlled trial | Yes |
|  | 2014 | NEJM | Goal-Directed Resuscitation for Patients with Early Septic Shock | Yes |
|  | 2010 | JAMA | Lactate clearance vs central venous oxygen saturation as goals of early sepsis therapy: a randomized clinical trial | Yes |
|  | 2018 | J Crit Care | P(v-a)CO_2_/C(a-v)O_2_-directed resuscitation does not improve prognosis compared with SvO2 in severe sepsis and septic shock: A prospective multicenter randomized controlled clinical study | No |
|  | 2015 | NEJM | Trial of Early, Goal-Directed Resuscitation for Septic Shock | No |
|  | 2017 | Crit Care | Use of stepwise lactate kinetics-oriented hemodynamic therapy could improve the clinical outcomes of patients with sepsis-associated hyperlactatemia | Yes |
| CQ5 | 2023 | N Engl J Med | Early Restrictive or Liberal Fluid Management for Sepsis-Induced Hypotension | No |
|  | 2019 | Crit Care Med | The Restrictive IV Fluid Trial in Severe Sepsis and Septic Shock (RIFTS): A Randomized Pilot Study | No |
|  | 2018 | Intensive Care Medicine | Restricting volumes of resuscitation fluid in adults with septic shock after initial  management: the CLASSIC randomised, parallel-group, multicentre feasibility trial | Yes |
|  | 2022 | ACADEMIC EMERGENCY MEDICINE | Restrictive fluids versus standard care in adults with sepsis in the emergency department (REFACED): A multicenter, randomized feasibility trial | Yes |
|  | 2018 | Intensive Care Med | Restricted fluid resuscitation in suspected sepsis associated hypotension (REFRESH): a pilot randomised controlled trial | Yes |
|  | 2022 | The New England Journal of Medicine | Restriction of Intravenous Fluid in ICU Patients with Septic Shock | No |
|  | 2020 | J Intensive Care Med. | Conservative Fluid Management after Sepsis Resuscitation: A Pilot Randomized Trial | Yes |
|  | 2015 | CHEST | Targeted Fluid Minimization Following Initial Resuscitation in Septic Shock A Pilot Study | No |

CQ: clinical question.

**Supplementary Table S3. List of outcome variables for data extraction**

| CQ | Outcome variables |
| --- | --- |
| CQ1 | # Short-term mortality (28-day mortality or 30-day mortality) |
|  | # Renal replacement therapy |
|  | # Hyperkalemia |
|  | # Mechanical ventilation |
|  | # Vasopressor |
|  | # Acute kidney injury |
|  | # Length of intensive care unit stay |
|  | # Short-term mortality (28-day mortality or 30-day mortality) |
| CQ2 | # Short-term mortality (approximately 28- day mortality) |
|  | # Severe adverse events (cardiovascular events, bowel ischemia, limb necrosis) |
|  | # Renal replacement therapy |
|  | # Length of intensive care unit stay |
| CQ3 | # Short-term mortality (28-day mortality or 30-day mortality) |
|  | # Organ failure |
|  | # Renal replacement therapy |
|  | # Length of vasopressor use or vasopressor-free days |
|  | # Length of intensive care unit stay |
|  | # Electrolyte abnormalities requiring therapeutic intervention |
| CQ4 | # Mortality (up to 90 days) |
|  | # Length of intensive care unit stay |
|  | # Intensive care unit mortality |
|  | # Ventilator-free days |
| CQ5 | # 90-day mortality |
|  | # Short-term mortality |
|  | # Severe acute kidney injury or renal replacement therapy |
|  | # Ventilator-free days |
|  | # Severe adverse events |
|  | # Vasopressor-free days |
|  | # Lung edema |
|  | # Amount of fluid administration |

CQ: clinical question

**Supplementary Table S4. Number of no, minor, and major errors in data extraction across large language models and prompt strategies in the first session**

| Category | LLM | Prompt strategy | Accuracy | | |
| --- | --- | --- | --- | --- | --- |
|  |  |  | No error | Minor error | Major error |
| Background | ChatGPT-4o | Original | 425 | 23 | 56 |
|  |  | Chain-of-thought | 411 | 33 | 60 |
|  |  | Self-reflection | 411 | 39 | 54 |
|  | Claude 3 Sonnet | Original | 471 | 9 | 24 |
|  |  | Chain-of-thought | 462 | 14 | 28 |
|  |  | Self-reflection | 477 | 7 | 20 |
|  | Gemini 1.5 Pro | Original | 452 | 8 | 44 |
|  |  | Chain-of-thought | 451 | 10 | 43 |
|  |  | Self-reflection | 467 | 6 | 31 |
| Outcome | ChatGPT-4o | Original | 133 | 31 | 44 |
|  |  | Chain-of-thought | 128 | 24 | 56 |
|  |  | Self-reflection | 119 | 37 | 52 |
|  | Claude 3 Sonnet | Original | 181 | 16 | 11 |
|  |  | Chain-of-thought | 172 | 23 | 13 |
|  |  | Self-reflection | 163 | 38 | 7 |
|  | Gemini 1.5 Pro | Original | 65 | 20 | 123 |
|  |  | Chain-of-thought | 74 | 13 | 121 |
|  |  | Self-reflection | 130 | 20 | 58 |

LLM: large language model.

**Supplementary Table S5. Number of no, minor, and major errors in data extraction across large language models and prompt strategies in the second session**

| Category | LLM | Prompt strategy | Accuracy | | |
| --- | --- | --- | --- | --- | --- |
|  |  |  | No error | Minor error | Major error |
| Background | ChatGPT-4o | Original | 416 | 35 | 53 |
|  |  | Chain-of-thought | 414 | 31 | 59 |
|  |  | Self-reflection | 424 | 32 | 48 |
|  | Claude 3 Sonnet | Original | 466 | 12 | 26 |
|  |  | Chain-of-thought | 469 | 9 | 26 |
|  |  | Self-reflection | 472 | 13 | 19 |
|  | Gemini 1.5 Pro | Original | 456 | 9 | 39 |
|  |  | Chain-of-thought | 453 | 12 | 39 |
|  |  | Self-reflection | 459 | 12 | 33 |
| Outcome | ChatGPT-4o | Original | 121 | 42 | 45 |
|  |  | Chain-of-thought | 136 | 29 | 43 |
|  |  | Self-reflection | 149 | 23 | 36 |
|  | Claude 3 Sonnet | Original | 144 | 54 | 10 |
|  |  | Chain-of-thought | 150 | 49 | 9 |
|  |  | Self-reflection | 176 | 25 | 7 |
|  | Gemini 1.5 Pro | Original | 61 | 26 | 121 |
|  |  | Chain-of-thought | 82 | 13 | 113 |
|  |  | Self-reflection | 123 | 31 | 54 |

LLM: large language model.

**Supplementary Table S6. Number of no, minor, and major errors in data extraction across large language models and prompt strategies in the third session**

| Category | LLM | Prompt strategy | Accuracy | | |
| --- | --- | --- | --- | --- | --- |
|  |  |  | No error | Minor error | Major error |
| Background | ChatGPT-4o | Original | 401 | 44 | 59 |
|  |  | Chain-of-thought | 417 | 40 | 47 |
|  |  | Self-reflection | 447 | 27 | 30 |
|  | Claude 3 Sonnet | Original | 463 | 16 | 25 |
|  |  | Chain-of-thought | 448 | 15 | 41 |
|  |  | Self-reflection | 470 | 10 | 24 |
|  | Gemini 1.5 Pro | Original | 459 | 12 | 33 |
|  |  | Chain-of-thought | 445 | 11 | 48 |
|  |  | Self-reflection | 458 | 12 | 34 |
| Outcome | ChatGPT-4o | Original | 133 | 41 | 34 |
|  |  | Chain-of-thought | 128 | 28 | 52 |
|  |  | Self-reflection | 131 | 34 | 43 |
|  | Claude 3 Sonnet | Original | 159 | 38 | 11 |
|  |  | Chain-of-thought | 165 | 36 | 7 |
|  |  | Self-reflection | 169 | 36 | 3 |
|  | Gemini 1.5 Pro | Original | 46 | 40 | 122 |
|  |  | Chain-of-thought | 55 | 19 | 134 |
|  |  | Self-reflection | 106 | 53 | 49 |

LLM: large language model.

**Supplementary Table S7. Types of errors in data extraction in the second session**

| Category | LLM | Prompt strategy | Type of error | | | |
| --- | --- | --- | --- | --- | --- | --- |
|  |  |  | Missing | Incorrect | Fabricated | Others |
| Background | ChatGPT-4o | Original | 69 | 10 | 0 | 9 |
|  |  | Chain-of-thought | 73 | 11 | 0 | 6 |
|  |  | Self-reflection | 54 | 10 | 5 | 11 |
|  | Claude 3 Sonnet | Original | 26 | 12 | 0 | 0 |
|  |  | Chain-of-thought | 19 | 14 | 0 | 2 |
|  |  | Self-reflection | 13 | 18 | 0 | 1 |
|  | Gemini 1.5 Pro | Original | 17 | 22 | 7 | 2 |
|  |  | Chain-of-thought | 19 | 21 | 9 | 2 |
|  |  | Self-reflection | 15 | 19 | 9 | 2 |
| Outcome | ChatGPT-4o | Original | 73 | 10 | 1 | 3 |
|  |  | Chain-of-thought | 65 | 6 | 1 | 0 |
|  |  | Self-reflection | 54 | 5 | 0 | 0 |
|  | Claude 3 Sonnet | Original | 60 | 3 | 1 | 0 |
|  |  | Chain-of-thought | 50 | 4 | 0 | 4 |
|  |  | Self-reflection | 28 | 1 | 1 | 2 |
|  | Gemini 1.5 Pro | Original | 122 | 3 | 20 | 2 |
|  |  | Chain-of-thought | 124 | 2 | 0 | 0 |
|  |  | Self-reflection | 58 | 7 | 20 | 0 |

LLM: large language model.

**Supplementary Table S8. Types of errors in data extraction in the third session**

| Category | LLM | Prompt strategy | Type of error | | | |
| --- | --- | --- | --- | --- | --- | --- |
|  |  |  | Missing | Incorrect | Fabricated | Others |
| Background | ChatGPT-4o | Original | 71 | 18 | 5 | 9 |
|  |  | Chain-of-thought | 65 | 15 | 0 | 7 |
|  |  | Self-reflection | 40 | 12 | 0 | 5 |
|  | Claude 3 Sonnet | Original | 24 | 16 | 0 | 1 |
|  |  | Chain-of-thought | 37 | 17 | 0 | 2 |
|  |  | Self-reflection | 14 | 20 | 0 | 0 |
|  | Gemini 1.5 Pro | Original | 16 | 19 | 8 | 2 |
|  |  | Chain-of-thought | 21 | 21 | 17 | 0 |
|  |  | Self-reflection | 16 | 20 | 8 | 2 |
| Outcome | ChatGPT-4o | Original | 63 | 2 | 10 | 0 |
|  |  | Chain-of-thought | 70 | 0 | 8 | 2 |
|  |  | Self-reflection | 68 | 1 | 3 | 5 |
|  | Claude 3 Sonnet | Original | 45 | 2 | 2 | 0 |
|  |  | Chain-of-thought | 42 | 0 | 1 | 0 |
|  |  | Self-reflection | 38 | 0 | 0 | 1 |
|  | Gemini 1.5 Pro | Original | 160 | 0 | 2 | 0 |
|  |  | Chain-of-thought | 151 | 0 | 2 | 0 |
|  |  | Self-reflection | 93 | 2 | 4 | 3 |

LLM: large language model.

**Supplementary Figure S1. Proportion of no, minor, and major errors in data extraction across large language models and prompt strategies in the first session**

**
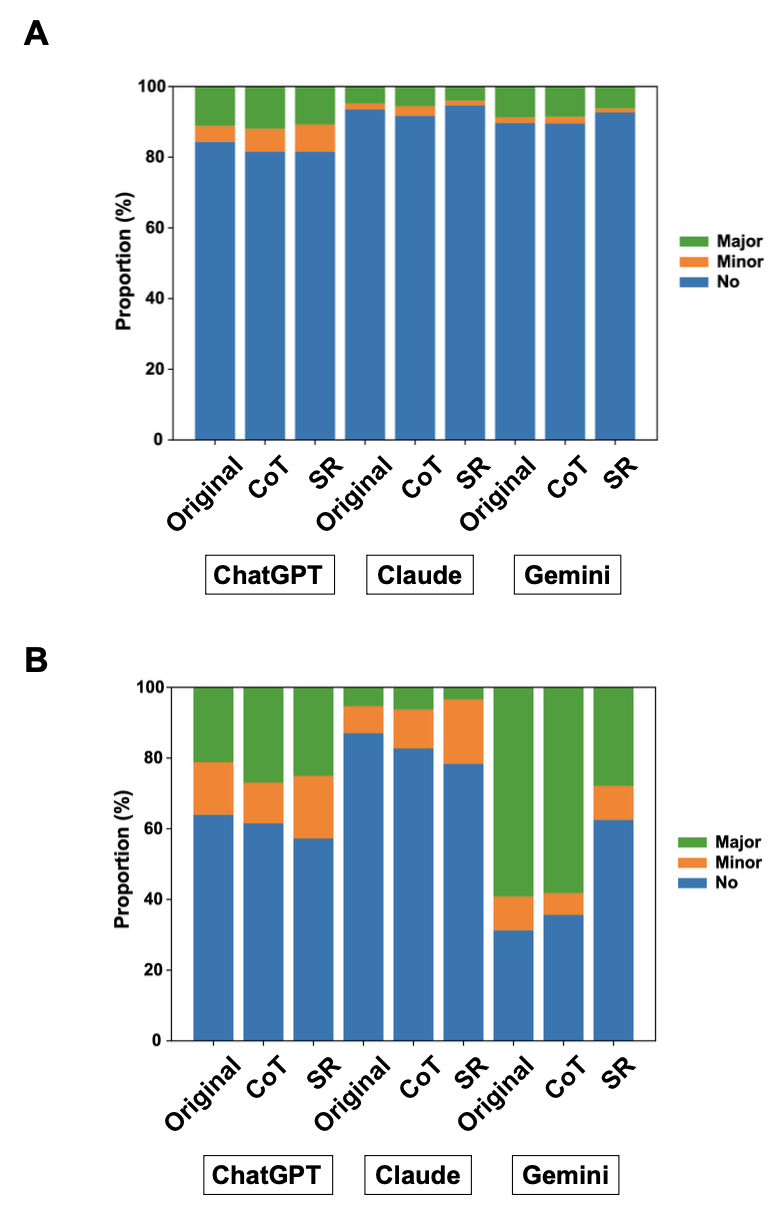
**

Proportion of accurate extractions across large language models and prompt strategies. Stacked bar charts summarize the proportion of no‑error (green), minor‑error (orange) and major‑error (red) extractions across all sessions for background (top) and outcome (bottom) variables.

**Supplementary Figure S2. Proportion of no, minor, and major errors in data extraction across large language models and prompt strategies in the second session**

**
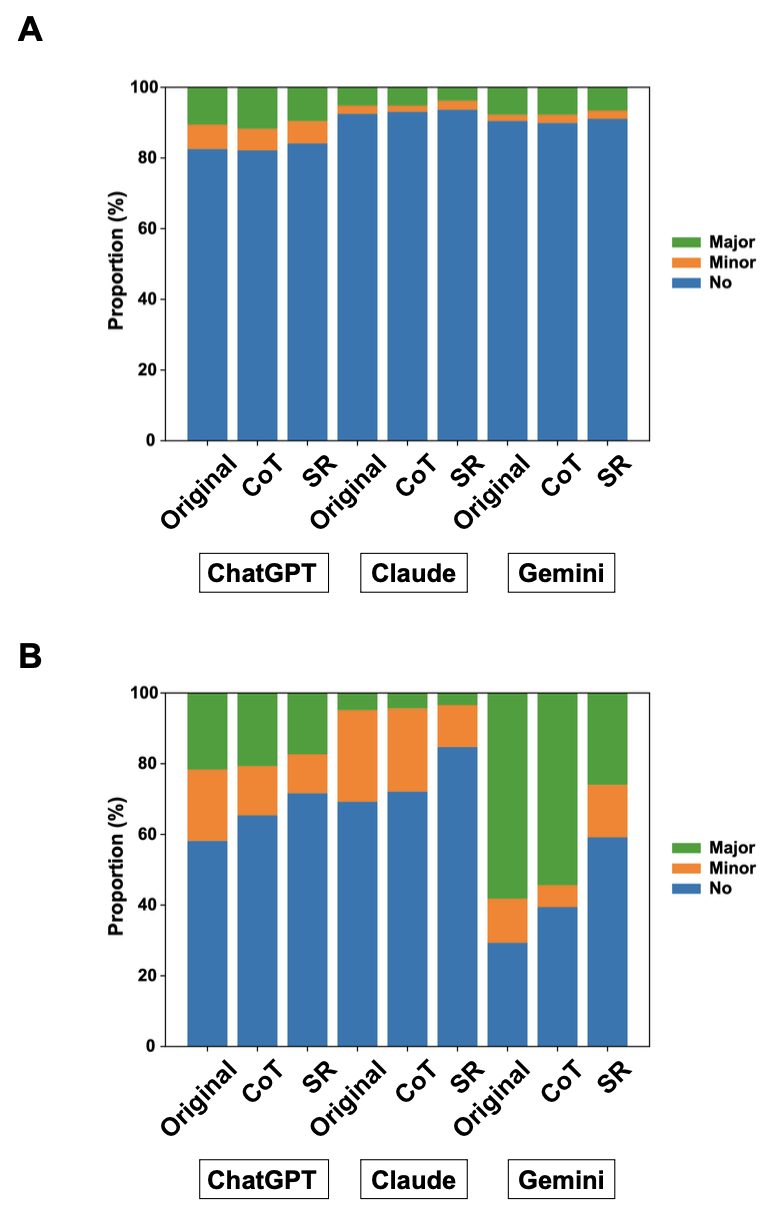
**

Proportion of accurate extractions across large language models and prompt strategies. Stacked bar charts summarize the proportion of no‑error (green), minor‑error (orange) and major‑error (red) extractions across all sessions for background (top) and outcome (bottom) variables.

**Supplementary Figure S3. Proportion of no, minor, and major errors in data extraction across large language models and prompt strategies in the third session**

**
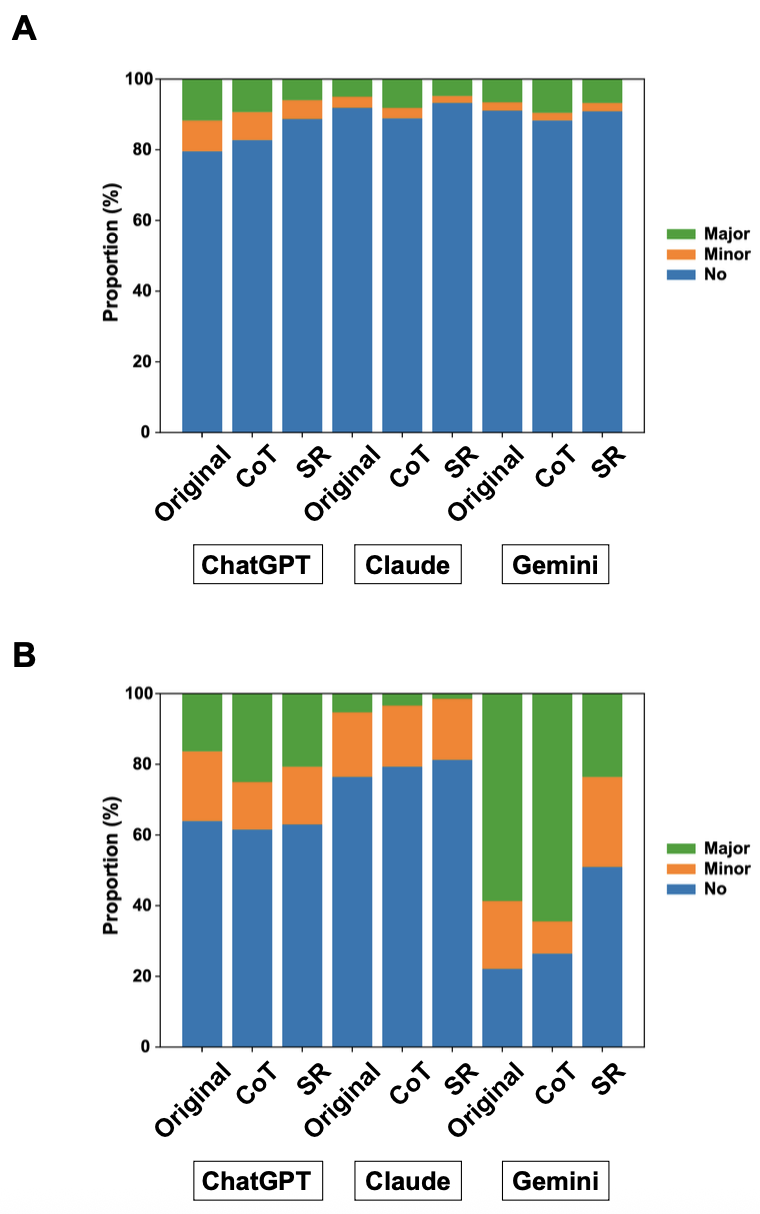
**

Proportion of accurate extractions across large language models and prompt strategies. Stacked bar charts summarize the proportion of no‑error (green), minor‑error (orange) and major‑error (red) extractions across all sessions for background (top) and outcome (bottom) variables.

**Supplementary Figure S4. Proportion of accuracy across large language models and prompt strategies**

**
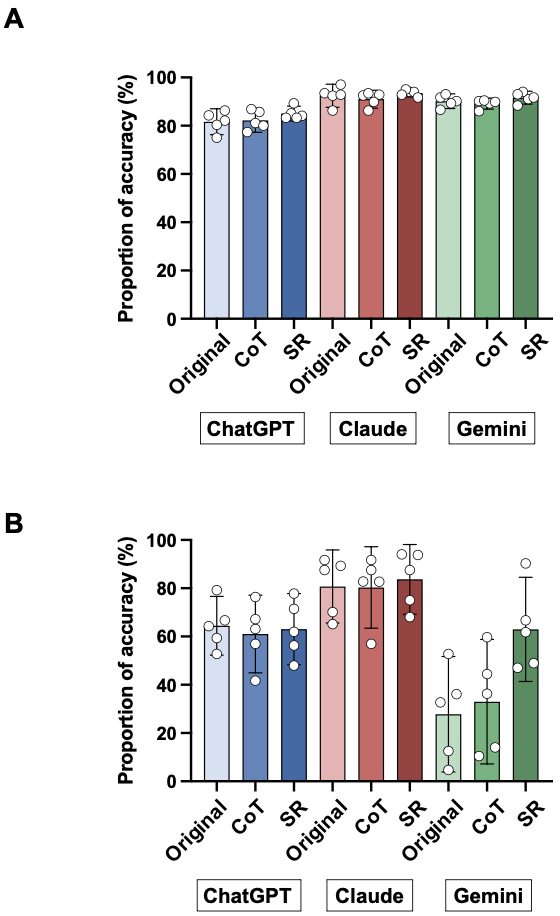
**

Proportion of accurate responses for extracting background (A) and outcome variables (B) across three large language models (ChatGPT, Claude, and Gemini) using three prompt designs: Original, chain-of-thought (CoT), and self-reflection (SR). Bars indicate the mean proportion of accuracy, and open circles represent individual clinical question.

**Supplementary Figure S5. Distribution of major and minor errors in background data extraction across large language models and prompt strategies**

**
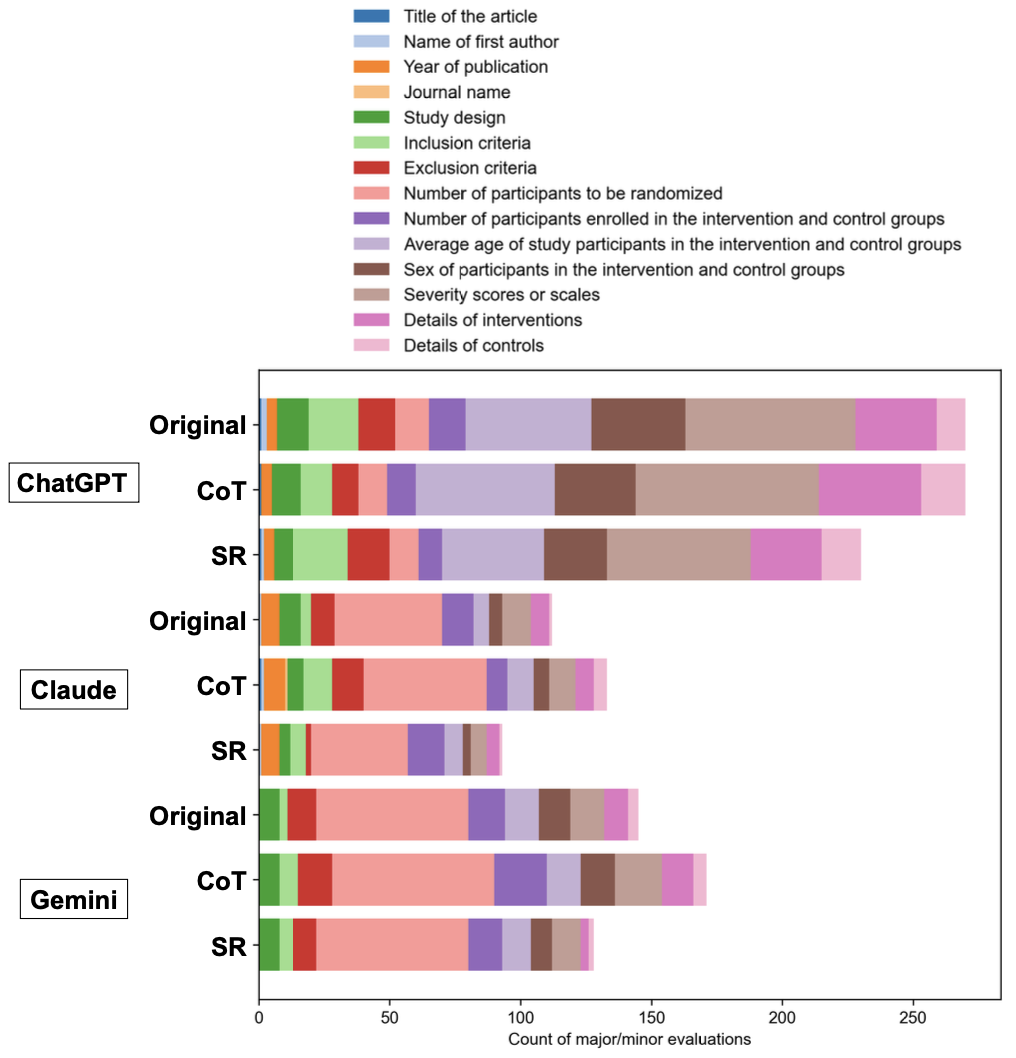
**

Horizontal stacked bars represent the cumulative counts of major and minor errors for each large language model (ChatGPT, Claude, and Gemini) and prompt strategy (original, chain-of-thought [CoT], and self-reflection [SR]), aggregated across three evaluation sessions. Each colored segment corresponds to a specific background data item extracted from the included studies.

**Supplementary Figure S6. Inter-rater agreement across large language models and prompt strategies in the first session**

**
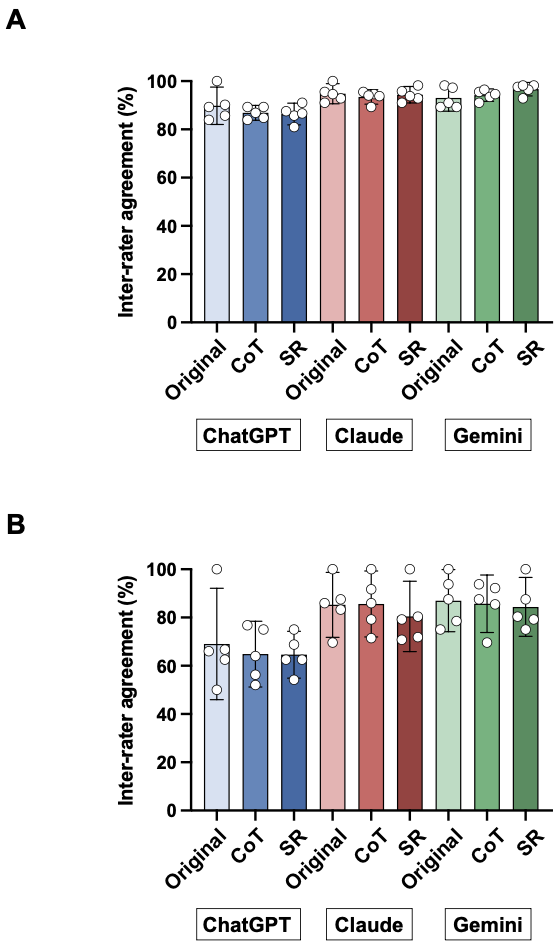
**

Inter-rater agreement between two human reviewers is expressed as the percentage of extraction cells for which both reviewers classified the model output identically (correct/incorrect). Bars indicate the mean proportion of agreement, and open circles represent individual clinical question. Each panel shows the agreement rate for extracting background (A) and outcome variables (B).CoT: Chain-of-thought; SR: Self-reflection.

**Supplementary Figure S7. Inter-rater agreement across large language models and prompt strategies in the second session**

**
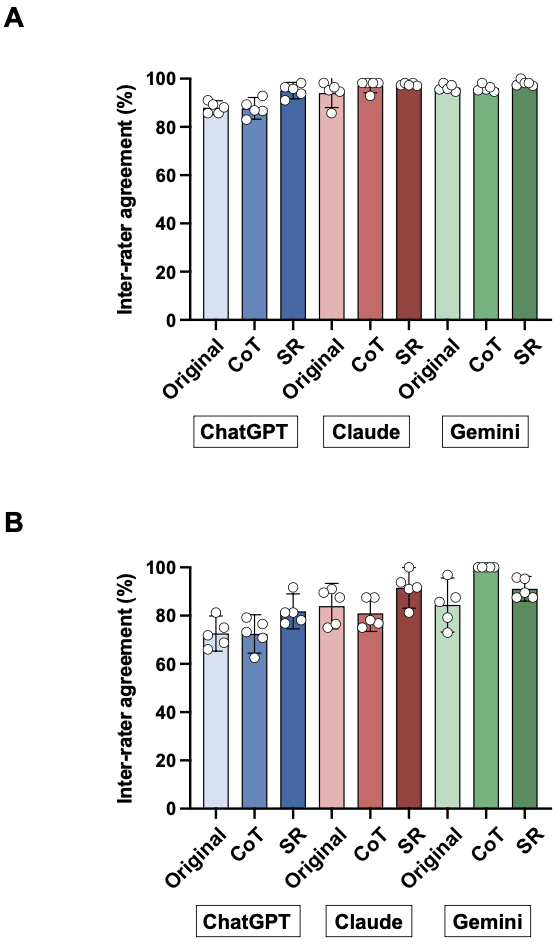
**

Inter-rater agreement between two human reviewers is expressed as the percentage of extraction cells for which both reviewers classified the model output identically (correct/incorrect). Bars indicate the mean proportion of agreement, and open circles represent individual clinical question. Each panel shows the agreement rate for extracting background (A) and outcome variables (B). CoT: Chain-of-thought; SR: Self-reflection.

**Supplementary Figure S8. Inter-rater agreement across large language models and prompt strategies in the third session**

**
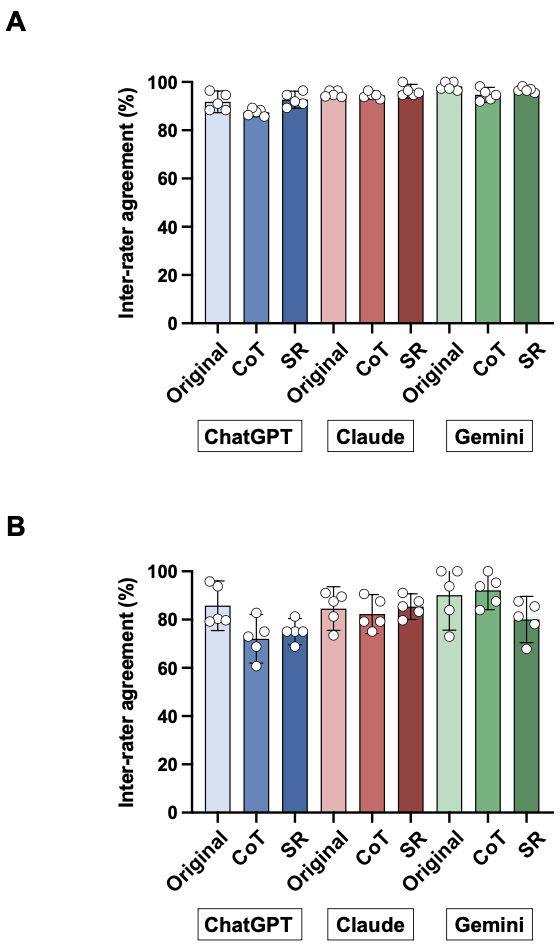
**

Inter-rater agreement between two human reviewers is expressed as the percentage of extraction cells for which both reviewers classified the model output identically (correct/incorrect). Bars indicate the mean proportion of agreement, and open circles represent individual clinical question. Each panel shows the agreement rate for extracting background (A) and outcome variables (B). CoT: Chain-of-thought; SR: Self-reflection.

**Supplementary Figure S9. Comparison of accuracy in data extraction in large language model-based data extraction between open access and non-open access publications**

**
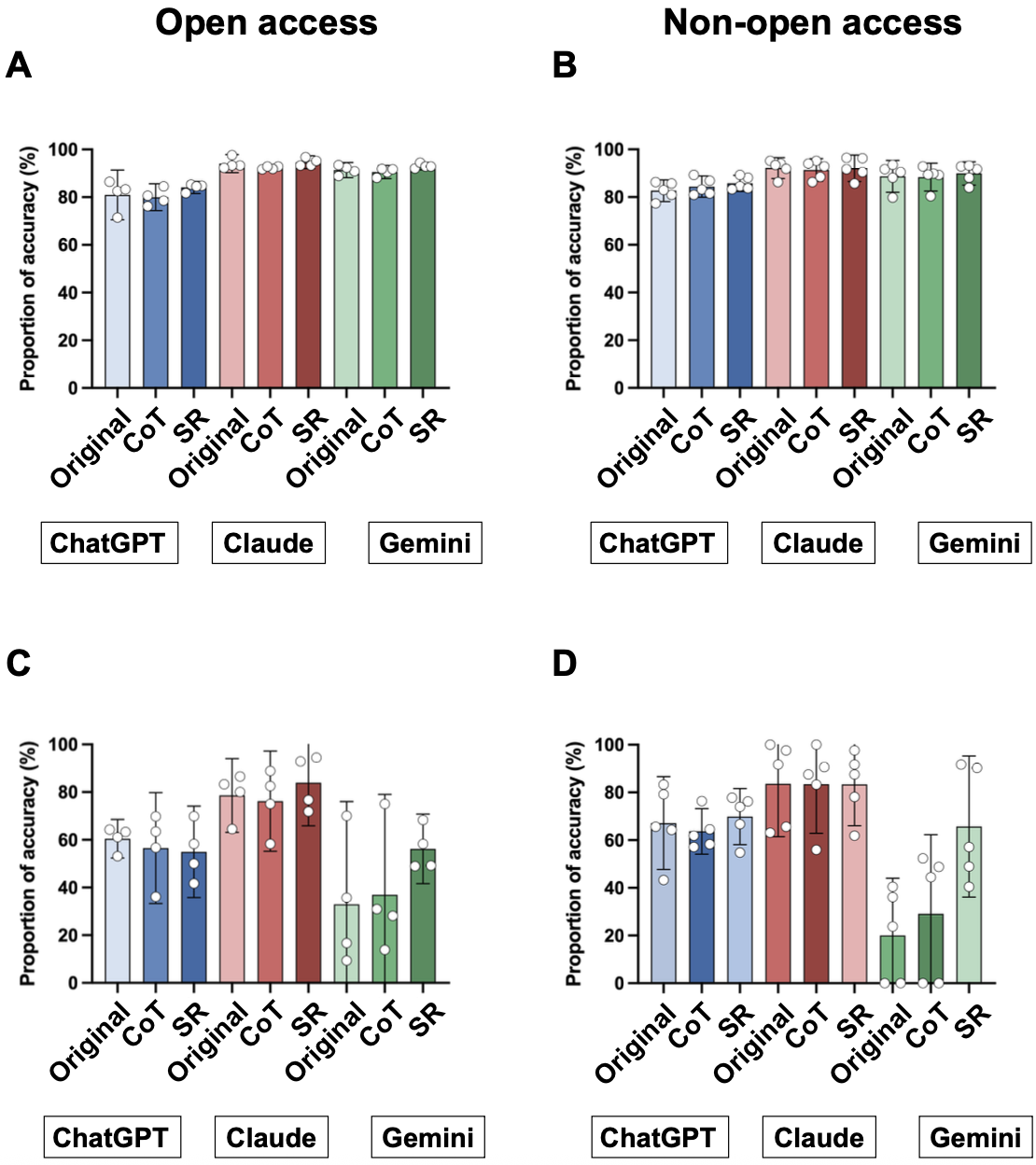
**

Comparison of no‑error proportions for extracting background (A, B) and outcome variables (C, D) between open‑access (A, C) and paywalled articles (B, D). Bars indicate the mean proportion of accuracy, and open circles represent individual clinical question. CoT: Chain-of-thought; SR: Self-reflection.
